# Comparison of the efficacy and safety of ultrasound-guided CHIVA and traditional HLS in the treatment of varicose veins of lower extremities – a meta-analysis

**DOI:** 10.1097/MD.0000000000035442

**Published:** 2023-11-03

**Authors:** Yueying Pei, Chuntao Li, Shuai Niu, Kun Jia, Fei Ju

**Affiliations:** a Department of Ultrasound, Hebei General Hospital, Shijiazhuang, China; b Department of Orthopedic, Shijiazhuang Second Hospital, Shijiazhuang, China; c Department of Vascular Surgery, Hebei General Hospital, Shijiazhuang, China; d Department of General Surgery, Shijiazhuang Hospital of traditional Chinese Medicine, Shijiazhuang, China.

**Keywords:** CHIVA, HLS, meta-analysis, ultrasound guidance, varicose veins of lower extremities

## Abstract

**Objective:**

Systematic evaluation of the efficacy and safety of conservative hemodynamic cure for venous insufficiency (CHIVA) compared with high ligation and stripping (HLS) in the treatment of varicose veins of lower extremities.

**Methods:**

We conducted a systematic literature search and compared the randomized controlled trial and retrospective cohort study of CHIVA and HLS in the treatment of varicose veins of lower extremities in several databases, including China National Knowledge Infrastructure, Wanfang database, cqvip datebase, PubMed, Cochrane library and EMBASE, to identify articles that might meet the criteria. Meta-analysis was performed using Revman 5.3 and Stata 13.0 software.

**Results:**

This Meta-analysis included a total of 14 research articles. This meta-analysis shows that CHIVA requires shorter operation time than HLS [mean difference (MD) = −13.57, 95% confidence interval (CI) (−21.05, −6.10), *P* = .0004]. There is less blood loss with CHIVA surgery [MD = −21.72, 95% CI (−30.35, −13.09), *P* < .00001]. The number of incisions made by the CHIVA technique is less [MD = −3.67, 95% CI (−4.03, −3.31), *P* < .00001]. Patients who underwent CHIVA had a shorter hospital stay [MD = −3.40, 95% CI (−4.72, −2.09), *P* < .00001]. The relapse rate was lower after CHIVA [OR = 0.36, 95% CI (0.18, 0.70), *P* = .003]. In terms of postoperative complications, CHIVA has a lower total complication rate [MD = 0.26, 95% CI (0.15, 0.46), *P* < .00001]. The incidence of deep vein thrombosis was lower after CHIVA [MD = 0.23, 95% CI (0.06, 0.92), *P* = .04]. CHIVA has a lower incidence of sensory disturbance than HLS [OR = 0.39, 95% CI (0.25, 0.60), *P* < .0001]. CHIVA technique has less nerve injury rate than HLS [OR = 0.11, 95% CI (0.02, 0.62), *P* = .01]. The incidence of hematoma was lower after CHIVA [OR = 0.48, 95% CI (0.27, 0.87), *P* = .02]. Among other metrics, the comparison results of the 2 techniques were similar.

**Conclusion:**

By comparison, it is found that CHIVA has shorter operation time, less blood loss, and fewer surgical incisions. Patients who underwent CHIVA surgery had shorter hospital stays and lower relapse rates. In terms of complications, the incidence of total complications after CHIVA is lower, and the incidence of postoperative deep vein thrombosis, postoperative sensory, nerve injury, and postoperative hematoma is also lower than that of HLS.

## 1. Background

Varicose veins of the lower extremities are one of the common diseases in vascular surgery, which causes discomfort such as swelling and numbness in the legs, and may also result in complications such as deep vein thrombosis (DVT) and venous ulcers, seriously affecting the quality of life of the patient.^[1]^ The most traditional surgical method for varicose veins of the lower extremities is high ligation and segmental stripping technique, but this surgery is highly traumatic with large skin scars and affects the appearance of the limb, with a high recurrence rate.^[2]^ In view of these shortcomings, there are also some other minimally invasive techniques currently available to treat this disease, such as foam sclerotherapy, intravenous and CHIVA (conservative hemodynamic cure for venous insufficiency) laser therapy. CHIVA technique was introduced by French scholar Franceschi 30 years ago.^[3]^ This technique represents a novel approach to treatment by surgically removing the refluxing saphenous and perforating veins, preserving some of the saphenous and main deep veins. Unlike HLS (high ligation and stripping) technique which destroys occlusive veins, CHIVA seals the reflux site and facilitates flow, providing a more precise treatment for varicose veins of the lower extremities.^[4]^ Translation: Thus, this study aims to analyze the advantages and disadvantages of the CHIVA and HLS techniques in the treatment of varicose veins of the lower extremities by comparing their efficacy.

## 2. Methods

### 2.1. Data sources and searches

Search for randomized controlled trials (RCT) and retrospective cohort studies (RCS) on HLS and CHIVA techniques for treating Varicose veins of lower extremities in China National Knowledge Infrastructure, WanFang datebas, PubMed, Embase, and Cochrane Library database. This study restricts the language to English and Chinese, and the time range of retrieval is set from the establishment of the database to December 2022. The keywords of this research retrieval include: Chronic Venous Disease; Ultrasound guidance; CHIVA; HLS; Varicose veins of lower extremities.

### 2.2. Inclusion criteria

1. Patients with primary varicose veins of lower extremities confirmed by ultrasound.
2. RCT and RCS comparing CHIVA with HLS therapy;
3. Clinical etiological anatomical pathophysiological rating is between C2 and C6 level.

### 2.3. Exclusion criteria

1. The patient is over 75 years old.
2. The test results suggest that the patients with severe abnormal blood coagulation and hematological diseases.
3. Patients with severe underlying diseases who cannot tolerate surgery.

Other reasons for exclusion include repeated studies, lack of access to the full text, case reports, cadaver studies, systematic review, incomplete data.

### 2.4. Data extraction and quality assessment

The following will be extracted from the study, including the author’s name, time of publication, country, type of study, number of patients, age, follow-up time, etc. The included RCTs will assess the risk of bias based on the tools provided by Cochrane Collaboration. The included RCS will assess the risk of bias based on Newcastle-Ottawa Scale. The evaluation includes 7 items: random sequence generation, allocation concealment, blinding of participants, blinding of outcome assessment, incomplete outcome date, selective reporting, and other bias. Each project is evaluated with “high,” “Low,” and “unclear.” If there are differences, they will be resolved through group discussion.

### 2.5. Statistical analysis

Meta-analysis was performed with Revman 5.3 and Stata 13.0 software. The counting data used odds ratio (OR) and 95% confidence interval (CI). The continuous data used mean difference (MD) and 95% CI. Chi-squared test and *I*^2^ were used to evaluate the heterogeneity. When *P* > 1 and *I*^2^>50%, the heterogeneity was small, and the fixed effect model was selected. If *P* < 1 and *I*^2^ > 50% indicate greater heterogeneity, the subgroup analysis is carried out according to different conditions, and the random effect model is selected.

### 2.6. Ethics approval statement

The study does not need to be approved by moral and ethical clerks.

## 3. Results

### 3.1. Search results

**Figure 1.**
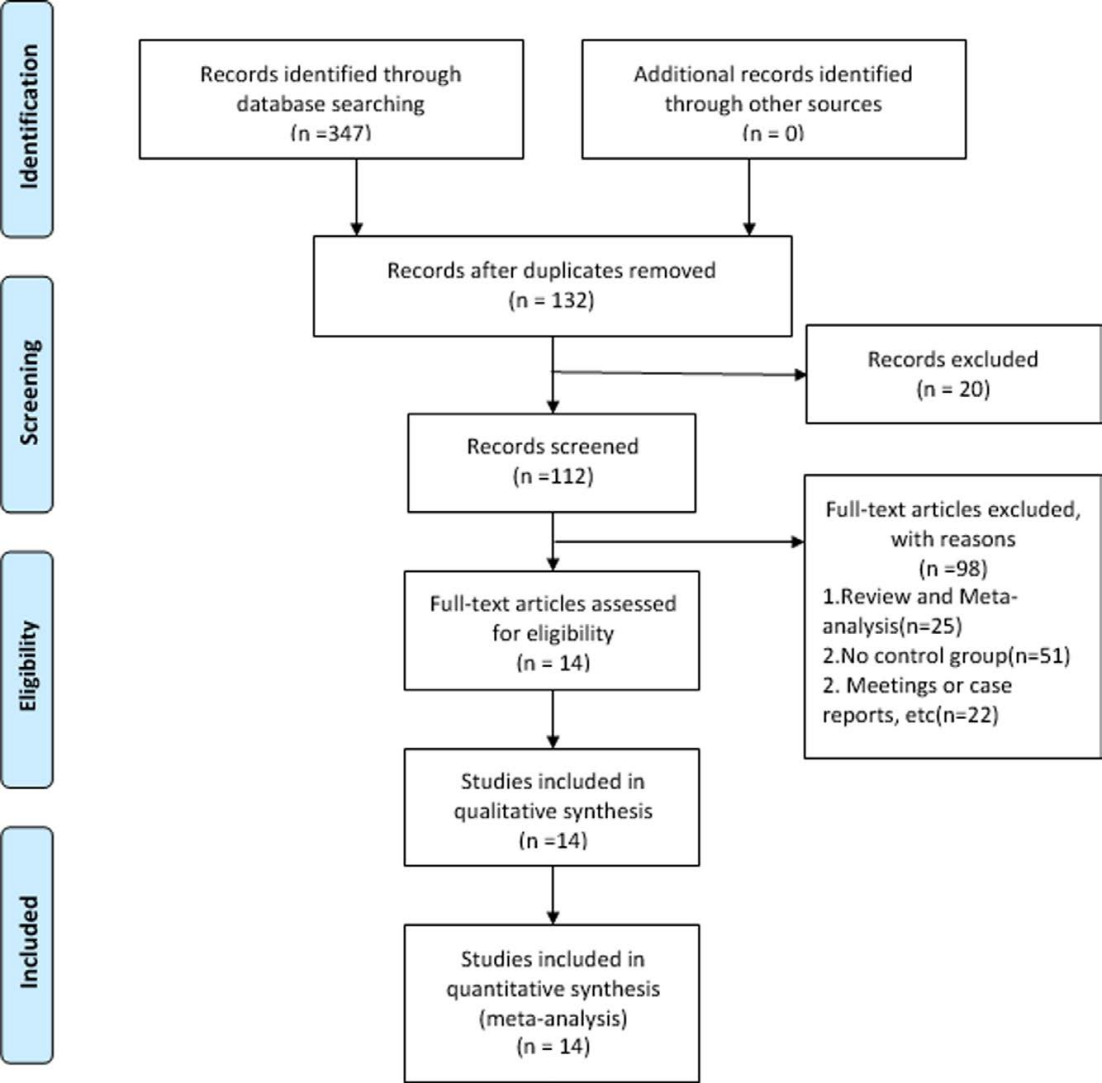
The flow chart of the literature screening process.

A total of 14 articles were found in these databases. Repeat and filter according to the set exclusion criteria, and articles that do not meet the requirements will be removed. Finally, a total of 14 articles were included in this study (Fig. 1).

### 3.2. Study characteristics and quality assessment

A total of 14 articles were included in this study, including 11 from China and 3 from Spain. A total of 2355 patients were included in the study, including 1023 patients with CHIVA and 1332 patients with HLS. The quality evaluation of the cohort study is shown in Table 1, and the randomized controlled trials is shown in Table 2.

**Table 1:** Baseline characteristics of included studies.

Author	Year	Country	Study design	Follow-up time	Number of patients (CHIVA/HLS)	Age	Course of disease	Outcomes	NOS Scores
Yuchun Xu	2021	China	RCT	48 mo	50/50	<60	3–27/2–28	①②③④⑤⑪⑫	8
Long Shi	2021	China	RCS	3 mo	23/82	58.21 ± 12.67/56.52 ± 9.60	–	①③④	7
Xiaohui Ma	2020	China	RCS	3 mo	188/226	58.4 ± 15.7/60.2 ± 16.3	–	①②③④⑥⑫	8
Bingxin Liu	2022	China	RCS	6 mo	57/61	54.86 ± 10.89/56.28/11.09	10.72 ± 5.67/12.92 ± 8.53	①②④⑤⑥⑦⑩⑫⑭⑮	8
Xiaoshuai Li	2022	China	RCS	24 mo	41/40	52.10 ± 6.44/51.15 ± 8.05	7.44 + 4.20/8.2 + 5.75	①②③④⑥⑦⑨⑪⑫⑭⑯	9
Bingshe Li	2018	China	RCS	12 mo	92/88	55 + 7/51 + 7	7.28 ± 2.43/7.44 ± 4.20	①②③④⑤⑨⑩⑬	7
Zeyu Guan	2021	China	RCS	12 mo	41/40	69.48 + 0.18/69.49 + 0.15	12.56 + 2.58/12.49 + 2.62	①③④⑧⑨⑩⑪⑮⑯	7
Qiuyan Chen	2021	China	RCT	12 mo	60/60	51.1 + 5.6/50.3 + 5.4	5.37 + 0.64/5.24 + 0.61	①②③④⑤⑧⑪⑫⑬⑮⑯	8
Yuan Chai	2021	China	RCS	6 mo	23/76	57.57 + 12.8/56.95 + 10.16	–	④⑦⑫⑭	7
Hua Wang	2016	China	RCT	12 mo	80/80	52.3 + 12.7/51.5 + 14.1	16.4 + 3.7/17.2 + 4.2	①③④⑤⑩⑪⑫⑬⑮⑯	7
Yong Hou	2020	China	RCS	6 mo	36/40	54.3 + 7.7/55.5 + 8.1	15.2 + 2.8/16.2 + 3.1	①②③④⑤	8
Jordi Maeso	2001	Spain	RCS	36 mo	90/85	50.9 ± 12.8/48.9 ± 11.1	–	④⑧⑫	7
Elena González Cañas	2020	Spain	RCT	48 mo	75/70	47.55 + 3/49.44 + 3	–	④⑩⑪⑫⑭⑯	7
Josep Oriol Pare´s	2010	Spain	RCT	5 yr	167/334	–	–	④⑧⑪	8

① = operation time, ② = blood loss ③ = hospital stays ④ = incidence of complications ⑤ = relapse rate ⑥ = number of surgical incisions ⑦ = VCSS score ⑧ = cure rate, ⑨ = blood flow velocity of superficial vein, ⑩ = incidence of DVT, ⑪ = postoperative infection rate, ⑫ = postoperative sensory disturbance, ⑬ = incidence of saphenous nerve injury, ⑭ = incidence of postoperative hematoma, ⑮ = incidence of superficial thrombophlebitis, ⑯ = incidence of ecchymosis.

NOS = Newcastle-Ottawa Scale.

**Table 2:** Risk of bias assessment of RCTs.

Author	Year	Country	Random sequence generation	Allocation concealment	Blinding of participants	Blinding of outcome assessment	Incomplete outcome date	Selective reporting	Other bias
Yuchun Xu	2021	China	Low risk	Low risk	Unclear	Unclear	Low risk	Low risk	Unclear
Qiuyan Chen	2021	China	Low risk	Low risk	Unclear	Unclear	Low risk	Low risk	Unclear
Hua Wang	2016	China	Low risk	Low risk	Low risk	Low risk	Low risk	Low risk	Unclear
Elena González Cañas	2020	Spain	Low risk	Low risk	High risk	Low risk	Low risk	Low risk	Unclear
Josep Oriol Pare’s	2010	Spain	Unclear	Unclear	High risk	Low risk	Low risk	Low risk	Unclear

RCT = randomized controlled trial.

### 3.3. Results of meta-analysis

#### 3.3.1. Operation time.

**Figure 2.**
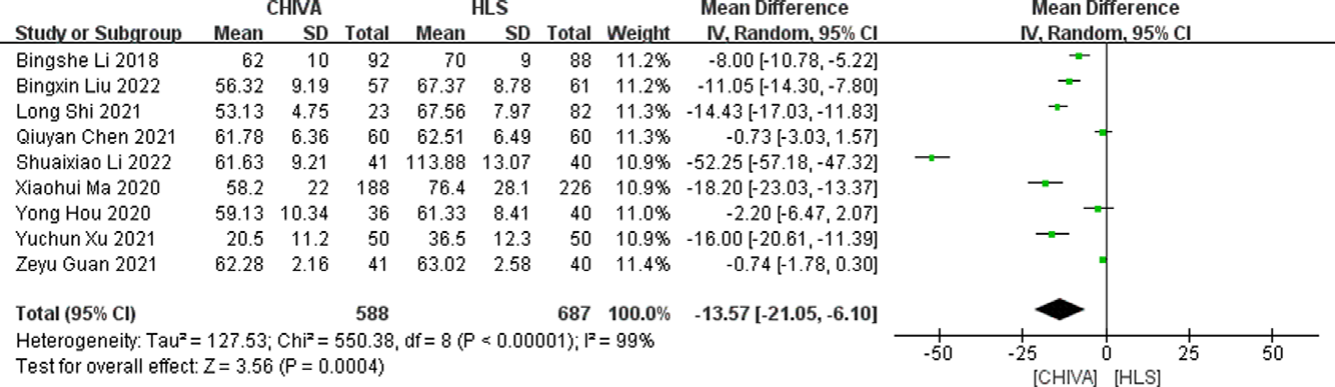
Forest plot of the comparison of the operation time between CHIVA and HLS. CHIVA = conservative hemodynamic cure for venous insufficiency, HLS = high ligation and stripping.

A total of 9 studies^[5–14]^ reported operation time, and the results of meta-analysis showed that there was a large heterogeneity between the studies (*I*^2^ = 99%), so the random effect model was used to combine the effect. The results of meta-analysis showed that CHIVA required less operation time [MD = −13.57, 95% CI (−21.05, −6.10), *P* = .0004] (Fig. 2).

#### 3.3.2. Blood loss.

**Figure 3.**
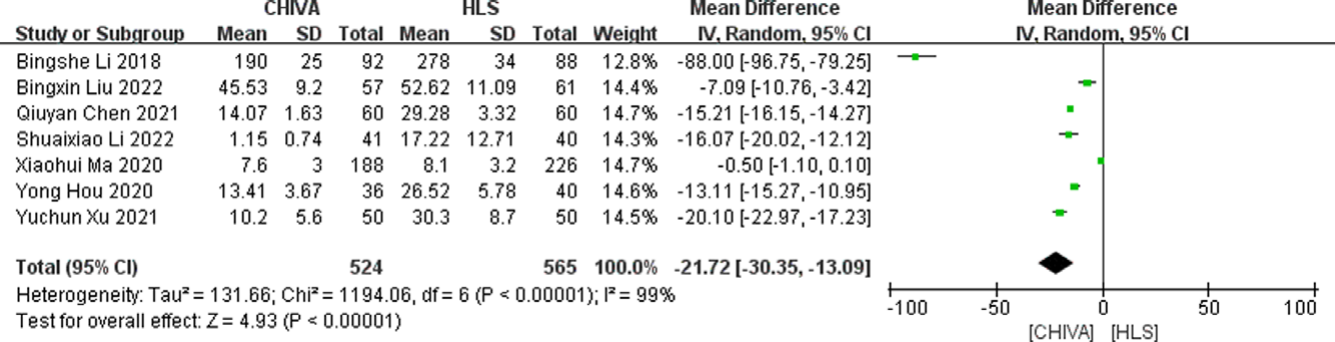
Forest plot of the comparison of the blood loss between CHIVA and HLS. CHIVA = conservative hemodynamic cure for venous insufficiency, HLS = high ligation and stripping.

A total of 7 studies^[6,7,9–13]^ reported blood loss, and the results of meta-analysis showed that there was a large heterogeneity between the studies (*I*^2^ = 99%), so the random effect model was used to combine the effect. The results of meta-analysis showed that CHIVA required less blood loss [MD = −21.72, 95% CI (−30.35, −13.09), *P* < .00001] (Fig. 3).

#### 3.3.3. Number of surgical incisions.

**Figure 4.**
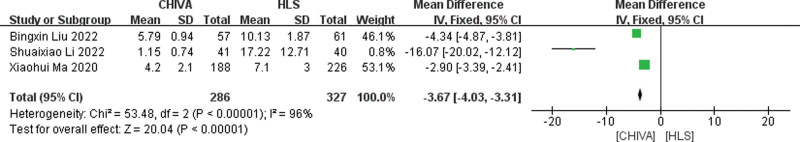
Forest plot of the comparison of the number of surgical incisions between CHIVA and HLS. CHIVA = conservative hemodynamic cure for venous insufficiency, HLS = high ligation and stripping.

A total of 3 studies^[7,10,11]^ reported number of surgical incisions, and the results of meta-analysis showed that there was a large heterogeneity between the studies (*I*^2^ = 96%), so the random effect model was used to combine the effect. The results of meta-analysis showed that the number of incisions in CHIVA operation is small [MD = −3.67, 95% CI (−4.03, −3.31), *P* < .00001] (Fig. 4).

#### 3.3.4. Hospital stays.

**Figure 5.**
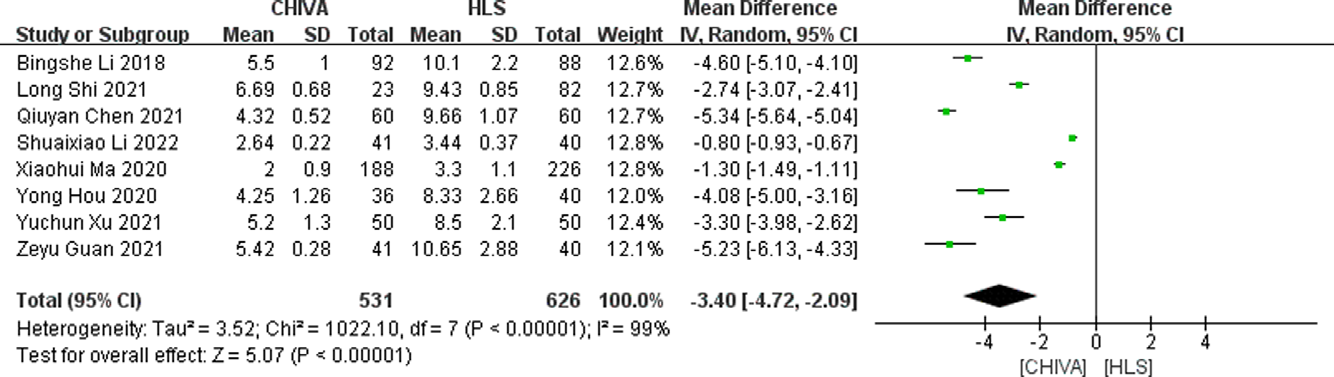
Forest plot of the comparison of hospital stays between CHIVA and HLS. CHIVA = conservative hemodynamic cure for venous insufficiency, HLS = high ligation and stripping.

A total of 8 studies^[6,8–14]^ reported length of stay in hospital, and the results of meta-analysis showed that there was a large heterogeneity between the studies (*I*^2^ = 99%), so the random effect model was used to combine the effect. The results of meta-analysis showed that the hospitalization time of patients after CHIVE is shorter [MD = −3.40, 95% CI (−4.72, −2.09), *P* < .00001] (Fig. 5).

#### 3.3.5. Cure rate.

**Figure 6.**
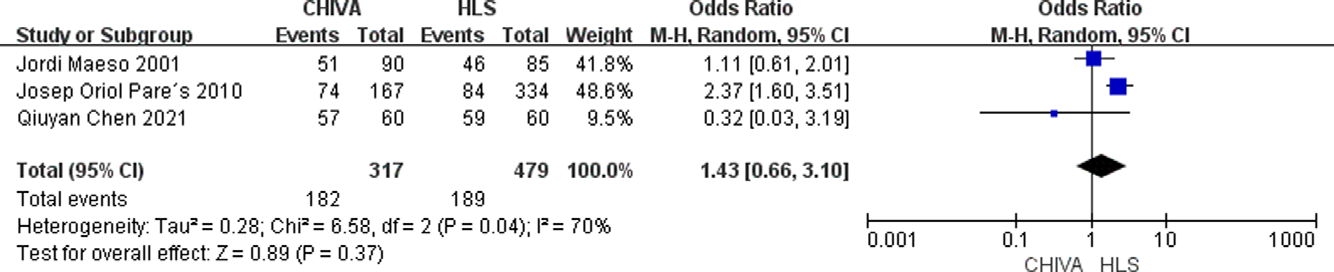
Forest plot of the comparison of cure rate between CHIVA and HLS. CHIVA = conservative hemodynamic cure for venous insufficiency, HLS = high ligation and stripping.

A total of 3 studies^[9,15,16]^ reported cure rate, and the results of meta-analysis showed that there was a large heterogeneity between the studies (*I*^2^ = 70%), so the random effect model was used to combine the effect. The results of meta-analysis showed that There was no significant difference in cure rate between CHIVE and HLS [MD = 1.43, 95% CI (0.66, 3.10), *P* = .37] (Fig. 6).

#### 3.3.6. Relapse rate.

**Figure 7.**
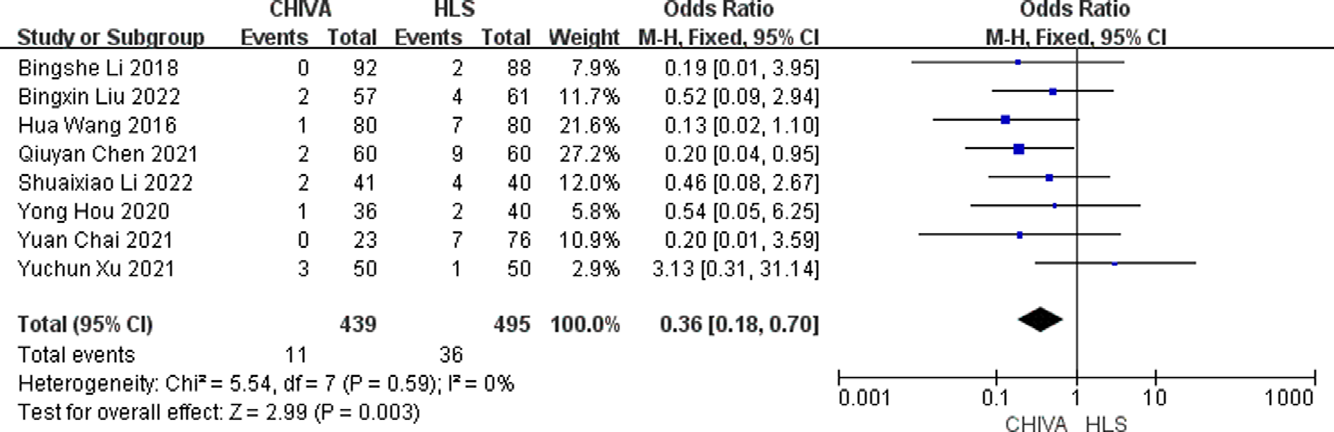
Forest plot of the comparison of relapse rate between CHIVA and HLS. CHIVA = conservative hemodynamic cure for venous insufficiency, HLS = high ligation and stripping.

A total of 8 studies^[6,7,9,11–13,17,18]^ reported relapse rate, and the results of meta-analysis showed that there was a small heterogeneity between the studies (*I*^2^ = 0%), so the fixed effect model was used to combine the effect. The results of meta-analysis showed that the relapse rate after CHIVA is lower [OR = 0.36, 95% CI (0.18, 0.70), *P* = .003] (Fig. 7).

#### 3.3.7. Venous clinical severity score.

**Figure 8.**
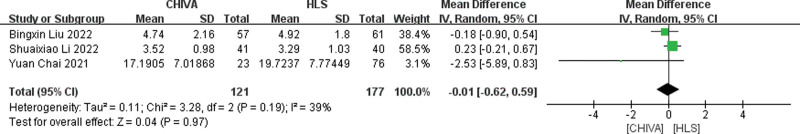
Forest plot of the comparison of VCSS score between CHIVA and HLS. CHIVA = conservative hemodynamic cure for venous insufficiency, HLS = high ligation and stripping, VCSS = venous clinical severity score.

A total of 3 studies^[7,11,18]^ reported venous clinical severity score (VCSS), and the results of meta-analysis showed that there was a small heterogeneity between the studies (*I*^2^ = 39%), so the fixed effect model was used to combine the effect. The results of meta-analysis showed that there was no significant difference in VCSS score after operation between CHIVE and HLS [MD = −0.01, 95% CI (−0.62, 0.59), *P* = .97] (Fig. 8).

#### 3.3.8. Blood flow velocity of superficial vein.

**Figure 9.**
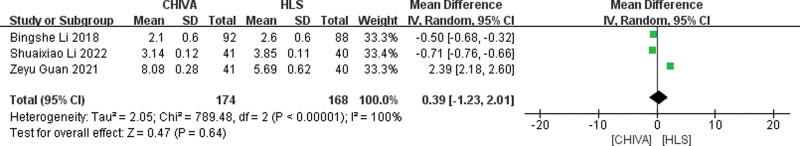
Forest plot of the comparison of blood flow velocity of superficial vein between CHIVA and HLS. CHIVA = conservative hemodynamic cure for venous insufficiency, HLS = high ligation and stripping.

A total of 3 studies^[6,11,14]^ reported blood flow velocity of superficial vein after operation, and the results of meta-analysis showed that there was a large heterogeneity between the studies (*I*^2^ = 100%), so the random effect model was used to combine the effect. The results of meta-analysis showed that there was no significant difference in Blood flow velocity of superficial vein after operation between CHIVE and HLS [MD = 0.39, 95% CI (−1.23, 2.01), *P* = .64] (Fig. 9).

#### 3.3.9. Toal complication rate.

**Figure 10.**
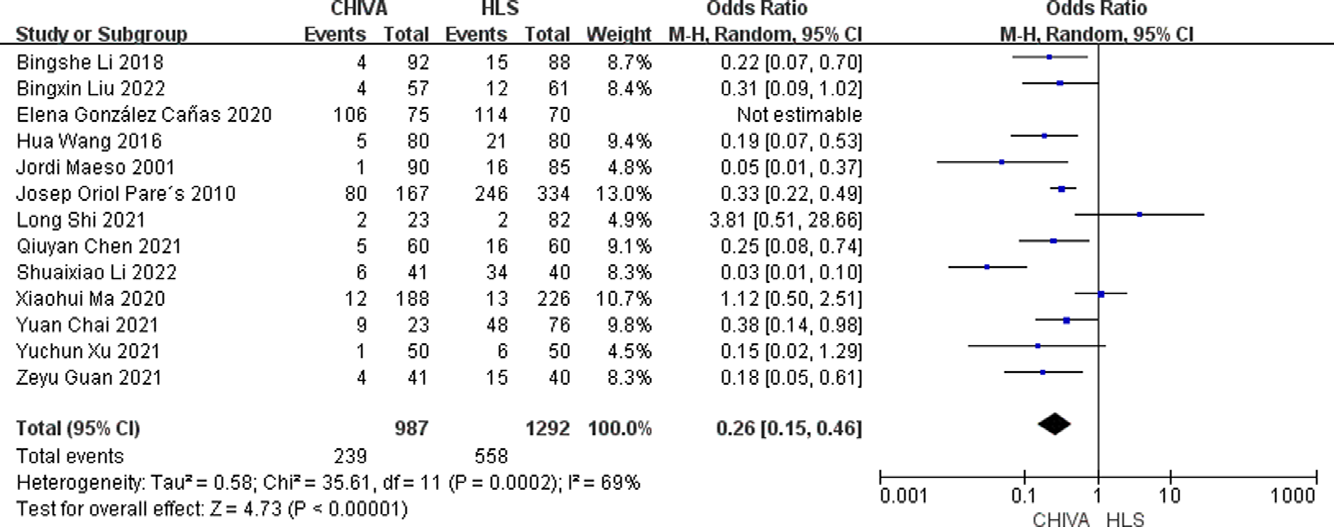
Forest plot of the comparison of incidence of complications between CHIVA and HLS. CHIVA = conservative hemodynamic cure for venous insufficiency, HLS = high ligation and stripping.

A total of 13 studies^[6–11,13–19]^ reported total complication rate, and the results of meta-analysis showed that there was a large heterogeneity between the studies (*I*^2^ = 69%), so the random effect model was used to combine the effect. The results of meta-analysis showed that the total complication rate of CHIVA operation is lower [MD = 0.26, 95% CI (0.15, 0.46), *P* < .00001] (Fig. 10).

### 3.4. Incidence of DVT

**Figure 11.**
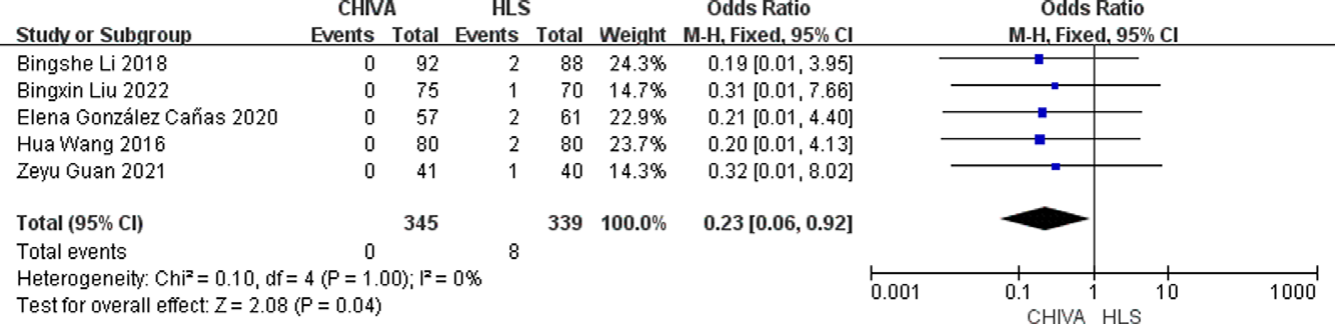
Forest plot of the comparison of incidence of DVT between CHIVA and HLS. CHIVA = conservative hemodynamic cure for venous insufficiency, DVT = deep vein thrombosis, HLS = high ligation and stripping.

A total of 5 studies^[6,7,14,17,19]^ reported Incidence of DVT, and the results of meta-analysis showed that there was a small heterogeneity between the studies (*I*^2^ = 0%), so the fixed effect model was used to combine the effect. The results of meta-analysis showed that the incidence of DVT after CHIVA is lower [MD = 0.23, 95% CI (0.06, 0.92), *P* = .04] (Fig. 11).

### 3.5. Postoperative infection rate

**Figure 12.**
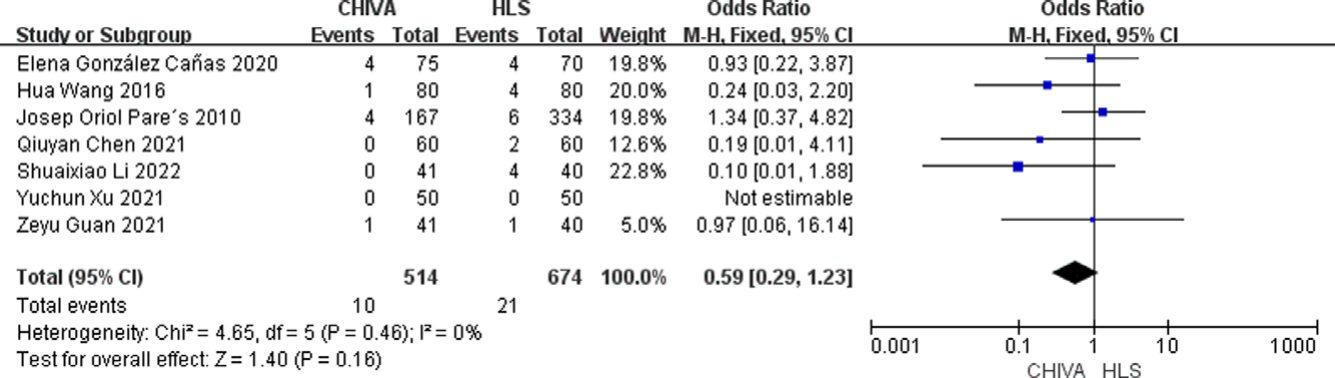
Forest plot comparing postoperative infection rates between CHIVA and HLS. CHIVA = conservative hemodynamic cure for venous insufficiency, HLS = high ligation and stripping.

A total of 7 studies^[10,11,13,14,16,17,19]^ reported postoperative infection rate, and the results of meta-analysis showed that there was a small heterogeneity between the studies (*I*^2^ = 0%), so the fixed effect model was used to combine the effect. The results of meta-analysis showed that There was no significant difference in postoperative infection rate between CHIVE and HLS [OR = 0.59, 95% CI (0.29, 1.23), *P* = .16] (Fig. 12).

### 3.6. Postoperative sensory disturbance

**Figure 13.**
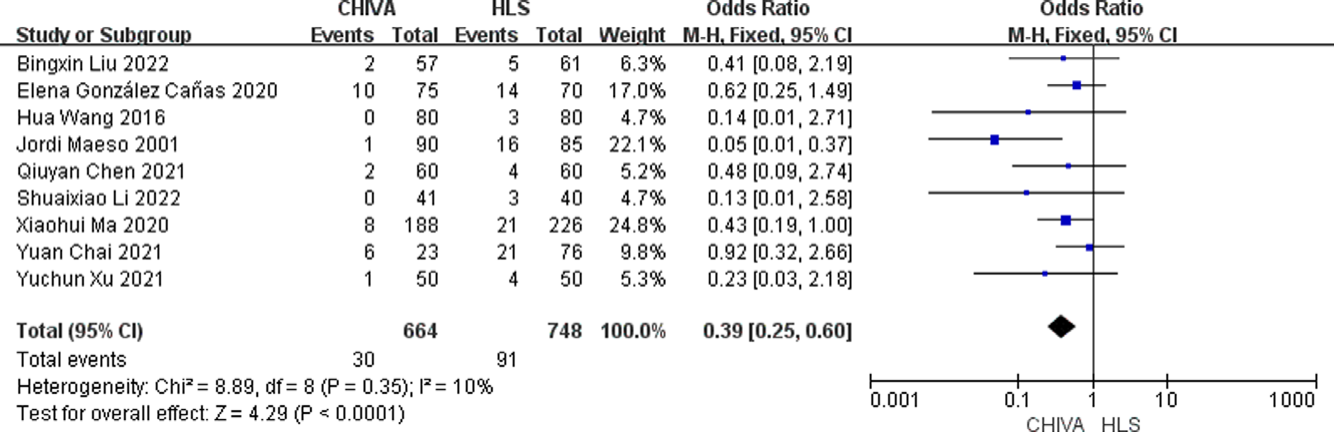
Forest plot comparing postoperative sensory disturbance between CHIVA and HLS. CHIVA = conservative hemodynamic cure for venous insufficiency, HLS = high ligation and stripping.

A total of 9 studies^[7,9–11,13,15,17–19]^ reported postoperative sensory disturbance, and the results of meta-analysis showed that there was a small heterogeneity between the studies (*I*^2^ = 10%), so the fixed effect model was used to combine the effect. The results of meta-analysis showed that the incidence of postoperative sensory disturbance after CHIVA is lower [OR = 0.39, 95% CI (0.25, 0.60), *P* < .0001] (Fig. 13).

### 3.7. Incidence of saphenous nerve injury

**Figure 14.**
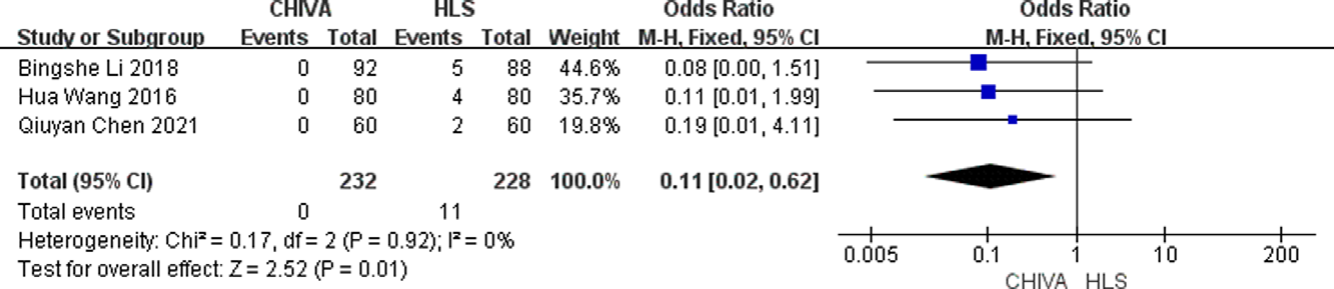
Forest plot comparing postoperative saphenous nerve injury between CHIVA and HLS. CHIVA = conservative hemodynamic cure for venous insufficiency, HLS = high ligation and stripping.

A total of 3 studies^[6,9,17]^ reported incidence of saphenous nerve injury, and the results of meta-analysis showed that there was a small heterogeneity between the studies (*I*^2^ = 0%), so the fixed effect model was used to combine the effect. The results of meta-analysis showed that the incidence of postoperative saphenous nerve injury after CHIVA is lower [OR = 0.11, 95% CI (0.02, 0.62), *P* = .01] (Fig. 14).

### 3.8. Incidence of postoperative hematoma

**Figure 15.**
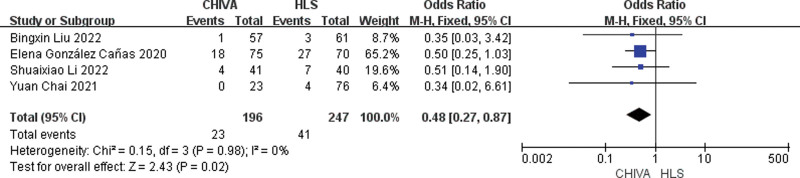
Forest plot comparing postoperative hematoma between CHIVA and HLS. CHIVA = conservative hemodynamic cure for venous insufficiency, HLS = high ligation and stripping.

A total of 4 studies^[7,11,18,19]^ reported incidence of postoperative hematoma, and the results of meta-analysis showed that there was a small heterogeneity between the studies (*I*^2^ = 0%), so the fixed effect model was used to combine the effect. The results of meta-analysis showed that the incidence of postoperative hematoma after CHIVA is lower [OR = 0.48, 95% CI (0.27, 0.87), *P* = .02] (Fig. 15).

### 3.9. Incidence of superficial thrombophlebitis

**Figure 16.**
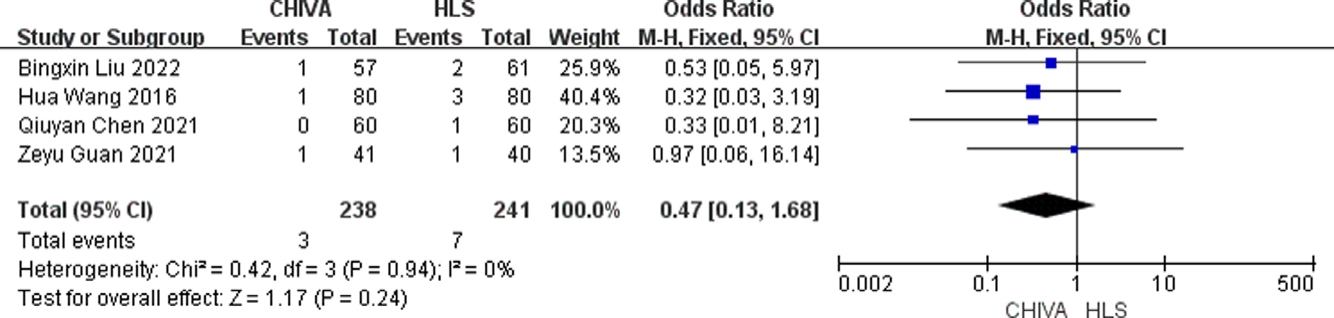
Forest plot comparing postoperative superficial thrombophlebitis between CHIVA and HLS. CHIVA = conservative hemodynamic cure for venous insufficiency, HLS = high ligation and stripping.

A total of 4 studies^[7,9,14,17]^ reported incidence of superficial thrombophlebitis, and the results of meta-analysis showed that there was a small heterogeneity between the studies (*I*^2^ = 0%), so the fixed effect model was used to combine the effect. The results of meta-analysis showed that There was no significant difference in superficial thrombophlebitis between CHIVE and HLS [OR = 0.47, 95% CI (0.13, 1.16), *P* = .24] (Fig. 16).

### 3.10. Incidence of ecchymosis

**Figure 17.**
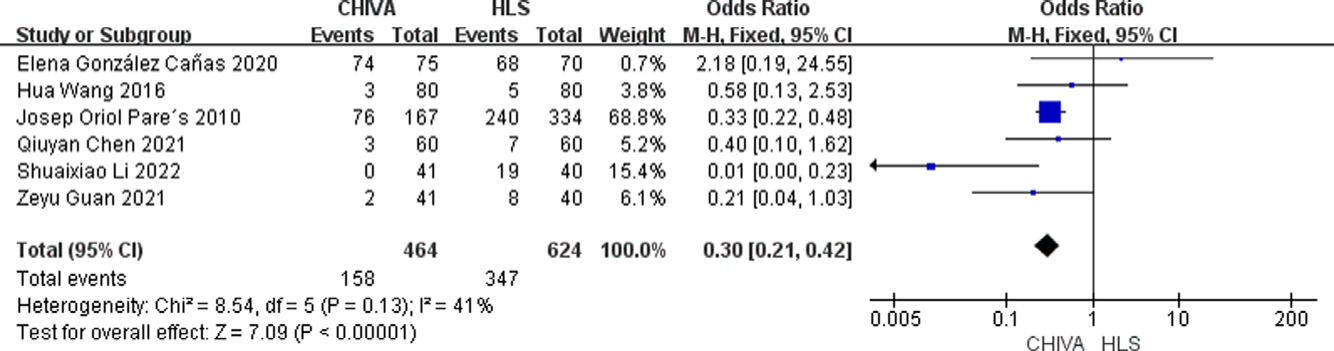
Forest plot comparing postoperative ecchymosis between CHIVA and HLS. CHIVA = conservative hemodynamic cure for venous insufficiency, HLS = high ligation and stripping.

A total of 6 studies^[9,11,14,16,17,19]^ reported incidence of ecchymosis, and the results of meta-analysis showed that there was a small heterogeneity between the studies (*I*^2^ = 41%), so the fixed effect model was used to combine the effect. The results of meta-analysis showed that the incidence of ecchymosis after CHIVA is lower [OR = 0.30, 95% CI (0.21, 0.42), *P* < .00001] (Fig. 17).

### 3.11. Sensitivity analyses and publication bias

**Figure 18.**
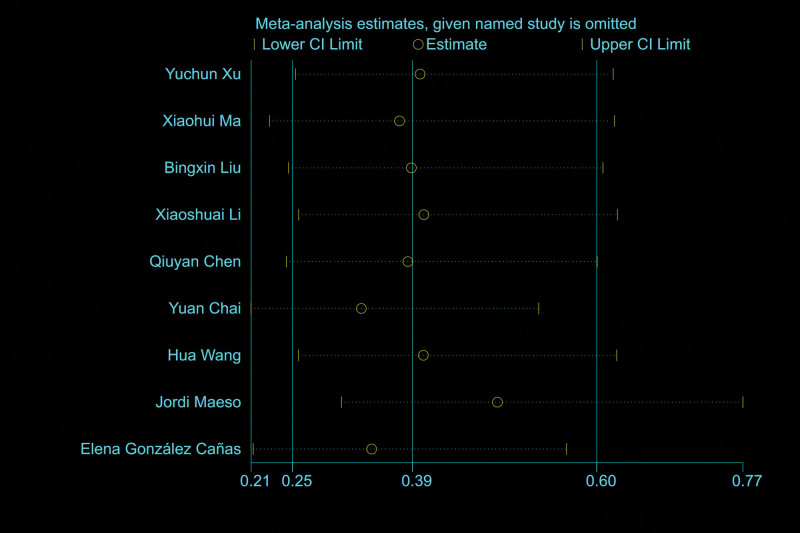
Sensitivity analyses by postoperative sensory disturbance.

**Figure 19.**
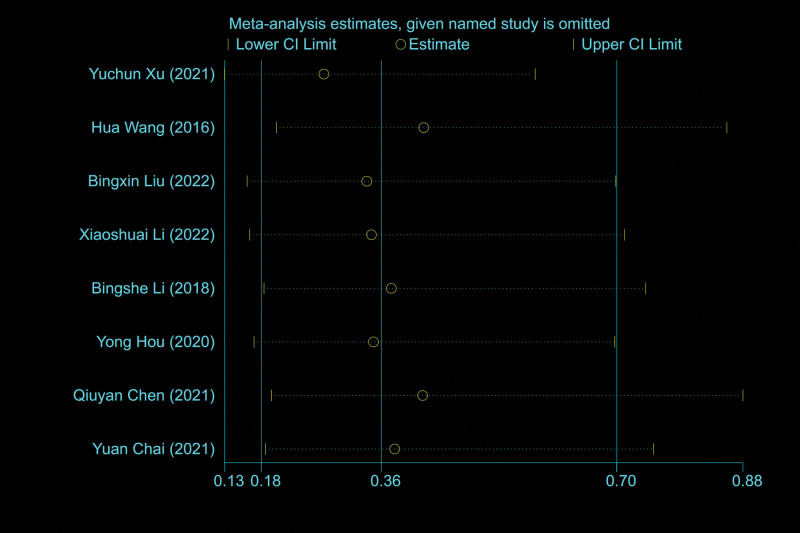
Sensitivity analyses by relapse rate.

Sensitivity analysis is conducted by omitting 1 study at a time to assess the robustness of our results. The sensitivity analyses show the stability of the results (Figs. 18 and 19).

## 4. Conclusion

Varicose veins of lower extremities is one of the common diseases in vascular surgery. At present, it is generally believed that the pathogenesis is blood reflux caused by femoral saphenous vein valvular insufficiency, and long-term venous hypertension produces symptoms such as limb heaviness, fatigue, acid distension, lower limb ulcers and so on.^[20]^ At present, there are many methods for the treatment of varicose veins, such as classical HLS techniques such as Endovenous laser ablation, Ultra sound guided foam sclerotherapy, CHIVA, etc. The new technique of CHIVA brings a new idea for the treatment of varicose veins of lower extremities. Under the guidance of ultrasound, this technique can provide accurate and individualized treatment for every patient. In the world, many scholars have reported the efficacy of CHIVA technology.^[21–23]^ However, compared with the classical HLS technology, which method is better is still controversial. Therefore, this time, through the form of meta-analysis, including a large number of studies, from the clinical effects, complications and other aspects of the 2 techniques for detailed analysis.

The evidence of this meta-analysis shows that CHIVA technology has significant advantages in terms of operation time, operative blood loss and the number of incisions. CHIVA technology can accurately locate the venous reflux point under the guidance of ultrasound, which greatly reduces the number of unnecessary incisions, shortens the time of operation and reduces the amount of bleeding. In addition, CHIVA technique only requires local anesthesia. After completing the operation under local anesthesia, patients can get out of bed without bed rest or special care, saving manpower and cost.^[7,13]^ In this meta-analysis, CHIVA technology showed lower recurrence rate and higher cure rate. The traditional HLS technique needs to peel off all the trunk of the great saphenous vein, resulting in the decrease of the blood vessels collecting the lower extremities and the increase of the pressure of the remaining blood vessels, resulting in the recurrence of varicose veins.^[24]^ In addition, large-scale stripping of blood vessels by HLS technology will produce a large amount of vascular endothelial growth factor, and the resulting neovascularization is connected with the superficial vein, which is more likely to cause varicose vein recurrence.^[25]^ When HLS technique is used to peel off the great saphenous vein, it will miss the perforating vein or excessively peel off the normal perforating vein. Through ultrasound guidance, CHIVA technology can identify the perforating veins that do not have normal drainage function, and accurately ligate the reflux point during the operation to block venous reflux, which is also one of the reasons for the low recurrence rate of CHIVA.^[26]^ In addition, the comparative results of VCSS scores also show the advantages of CHIVA in the therapeutic effect. In terms of superficial venous reflux velocity, it is also confirmed once again that CHIVA can retain the advantage of perforating veins and better improve the positive blood flow of lower extremity veins.^[27,28]^ In this meta-analysis, CHIVA technology showed some advantages in the incidence of complications. The overall incidence of complications of CHIVA was lower than that of HLS, and the incidence of DVT, postoperative sensory disturbance, hematoma and ecchymosis was lower than that of HLS. The main reason for this phenomenon is that CHIVA technology is guided by ultrasound, which is an accurate treatment to avoid nerve injury and bleeding caused by large-scale incisions of HLS technology. In terms of the incidence of infection, there was no significant difference between the two.

There are some limitations in this study. First of all, there are only 7 RCT articles included in this study, and the level of evidence may be insufficient. Secondly, in the included research, China is a country with a large number of articles, while other countries have a small number of articles, which may lead to regional bias.

## Author contributions

**Conceptualization:** Yueying Pei.

**Data curation:** Chuntao Li, Kun Jia, Fei Ju.

**Formal analysis:** Yueying Pei, Chuntao Li, Shuai Niu.

**Investigation:** Chuntao Li, Shuai Niu, Kun Jia.

**Methodology:** Chuntao Li.

**Project administration:** Yueying Pei.

**Resources:** Shuai Niu, Kun Jia.

**Software:** Chuntao Li.

**Supervision:** Yueying Pei.

**Validation:** Chuntao Li.

**Visualization:** Chuntao Li.

**Writing – original draft:** Yueying Pei, Chuntao Li.

**Writing – review & editing:** Yueying Pei.
